# 凡德他尼治疗5例晚期复治肺腺癌患者的临床观察及相关文献回顾

**DOI:** 10.3779/j.issn.1009-3419.2012.02.11

**Published:** 2012-02-20

**Authors:** 丽丽 郭, 俊舫 唐, 弃逸 孟, 允中 朱, 丽艳 徐, 鹤玲 史, 喆 刘

**Affiliations:** 101149 北京，首都医科大学附属北京胸科医院肿瘤内科 Department of Medical Oncology, Beijing Chest Hospital, Capital Medical University, Beijing 101149, China

**Keywords:** 肺肿瘤, 凡德他尼, EGFR-TKIs, Lung neoplasms, Vandetanib, EGFR-TKIs

## Abstract

**背景与目的:**

凡德他尼是一种多靶点口服的小分子抑制剂，可同时作用于血管内皮生长因子受体、表皮生长因子受体、RET酪氨酸激酶转染中的重排，本研究旨在探讨凡德他尼治疗晚期复治肺腺癌患者的疗效和副反应。

**方法:**

患者经过化疗和特罗凯治疗失败后，给予凡德他尼300 mg每日1次口服。

**结果:**

5例患者中，2例（40%）最佳疗效达疾病稳定（stable disease, SD），3例（60%）疗效均为疾病进展（progressive disease, PD）。随访40个月，1例患者目前仍在随访中。中位无疾病进展时间（progression free survival, PFS）为2个月，平均总生存期（overall survival, OS）为22.6个月。出现副反应包括皮疹（*n*=2）、皮肤改变（*n*=2）、甲沟炎（*n*=2）、无症状的心电图QTc延长（*n*=2）、ST-T改变（*n*=1）、腹泻（*n*=1）、转氨酶增高（*n*=1）。

**结论:**

凡德他尼治疗晚期复治肺腺癌患者中位PFS为2个月，平均OS为22.6个月，具有较好的安全性，结果同相关文献报道类似。

## Abstract (en)

**Background and objective:**

Vandetanib is a once-daily oral multi-target inhibitor of vascular endothelial growth factor receptor, epidermal growth factor receptor, and rearranged during transfection (RET) tyrosine kinases. The current study aimed to evaluate the effect and safety of vandetanib administered in refractory advanced lung adenocarcinoma patients.

**Methods:**

Five patients who accepted chemotherapy and Tarceva therapy as first- and second-line treatments received vandetanib (300 mg, oral, once daily).

**Results:**

The effects are stable disease on two patients (40%) and progressive disease on three patients (60%). With a median follow-up of 36 months, one patient remained on follow-up. The median progression free survival (PFS) is 2 months, and the mean overall survival is 22.6 months. The adverse events include rash (*n*=2), skin change (*n*=2), paronychia (*n*=2), asymptomatic QTc prolongation (*n*=2), ST-T change (*n*=1), diarrhea (*n*=1), and increased transaminase (*n*=1).

**Conclusion:**

There were lower incidences of severe side effects with vandetanib therapy in refractory advanced lung adenocarcinoma patients. The results of effect and safety of vandetanib are similar with the related reviewed articles.

肺癌是全世界范围导致癌症死亡的主要原因，大部分患者确诊时已经处于晚期，虽然化疗仍是晚期非小细胞肺癌（non-small cell lung cancer, NSCLC）治疗的基石，但目前的治疗似乎到了一个平台期。近年来，随着分子生物学的发展和靶向治疗的开展，在NSCLC治疗基本清楚的几种传导通路中，针对血管内皮生长因子（vascular endothelial growth factor, VEGF）、表皮生长因子受体（endothelia growth factor receptor, EGFR）通路的靶向药物在肺癌治疗中的地位越来越重要^[1-3]^。

多靶点药物通过对多种路径的抑制起到协同抗肿瘤作用。凡德他尼是一个口服多靶点药物，通过抑制VEGF、EGFR、RET酪氨酸激酶转染中的重排过程中不同的细胞内信号传导路径，抑制肿瘤细胞的生长和血管生成。前期研究^[4-6]^显示其具有抗肿瘤活性和较低的毒性。通过对*RET*基因过表达的抑制，2011年4月美国食品药品监督管理局批准凡德他尼用于治疗成人晚期（转移型）不适合手术且疾病在持续发展的甲状腺髓样癌患者。本研究选择EGFR酪氨酸激酶抑制剂（EGFR-tyrosion kinase inhibitors, EGFR-TKIs）治疗后的肺腺癌患者，应用凡德他尼口服治疗，观察疗效和毒性反应。

## 1 对象与方法

### 1.1 对象

2008年8月-2008年12月北京胸科医院肿瘤内科住院患者，其中男性2例，女性3例，年龄在26岁-61岁之间，中位年龄45岁。既往经细胞学或组织学确诊的局部晚期或转移性肺腺癌（Ⅲb期Ⅳ期）；应用一线或二线化疗失败后，又经历特罗凯治疗后进展的患者；行螺旋CT扫描检查有客观可测量的病灶；病理类型均为腺癌，分期为Ⅳ期；患者的生活属地和种族无明显差异；ECOG PS评分在0分-2分，心、肝、肾功能正常（表 1）。

**1:** 入组病人基本人口学和临床资料 Baseline demographics and clinical characteristics of patients

	Gender	Age (year)	Efficacy	Chemotherapy regimen/ cycles	Chemotherapy PFS (month)	Tarceva PFS (month)	Vandetanib PFS (month)	OS (month)
1	Female	34	SD	GP1, NP1	15	22	7.5	Following
2	Female	48	SD	DC1, GC3	9	14	11	24
3	Male	45	PD	GC4	4	10	1.5	28
4	Male	26	PD	GC4	5	2	2	16
5	Female	61	PD	TC2	1.5	1	0.5	5
PFS: progression free survival; OS: overall survival; SD: stable disease; PD: progressive disease.								

### 1.2 方法

#### 1.2.1 治疗方案

研究药物来源于阿斯利康制药公司生产的凡德他尼，应用凡德他尼300 mg每日1次口服，持续到患者肿瘤疾病进展。该研究为阿斯利康公司进行的D4200C00044研究的一部分，患者均已签署知情同意书，符合伦理要求。

#### 1.2.2 疗效评价

按照RECIST 1.0评价标准进行评价。

#### 1.2.3 毒副反应

按照CTC 3.0毒副反应标准进行评价。

### 1.3 统计

生存时间按月计算，总共随访40个月。采用SPSS 13.0统计软件。

## 2 结果

### 2.1 疗效

5例患者中，男性2例，女性3例，其中2例（40%）最佳疗效达SD，均为女性患者；其他3例（60%）疗效均为PD。

### 2.2 生存期

随访40个月，其中4例患者已去世，1例患者目前仍在随访中。中位无疾病进展期（progression free survival, PFS）为2个月，中位总生存期（overall survival, OS）为22.6个月（图 1，图 2）。

**1:**
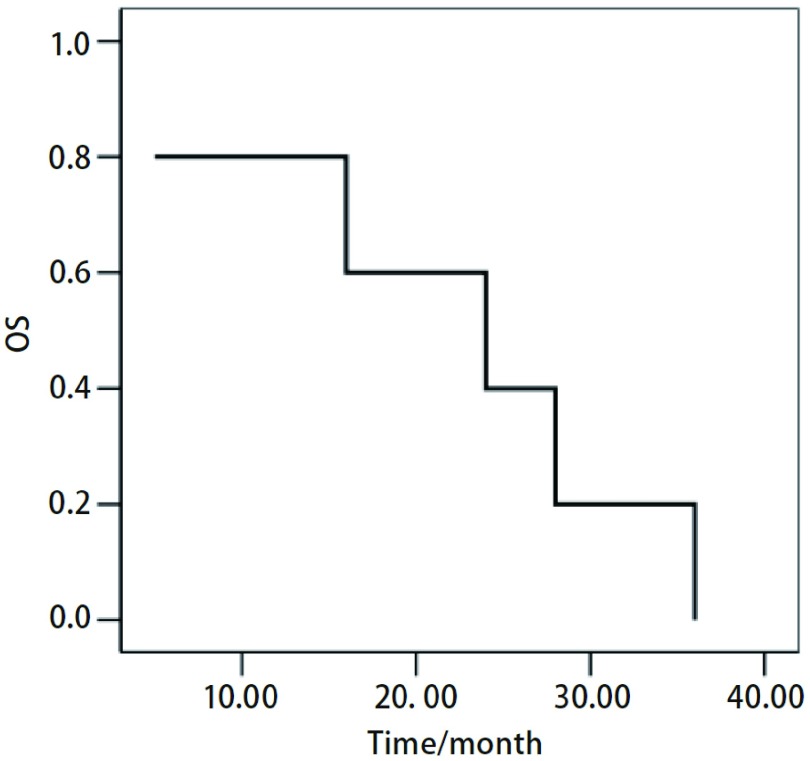
凡德他尼治疗肺腺癌患者的生存曲线 *Kaplan*-*Meier* curve for overall survival of lung adenocarcinoma patients treated by vandetanib

**2:**
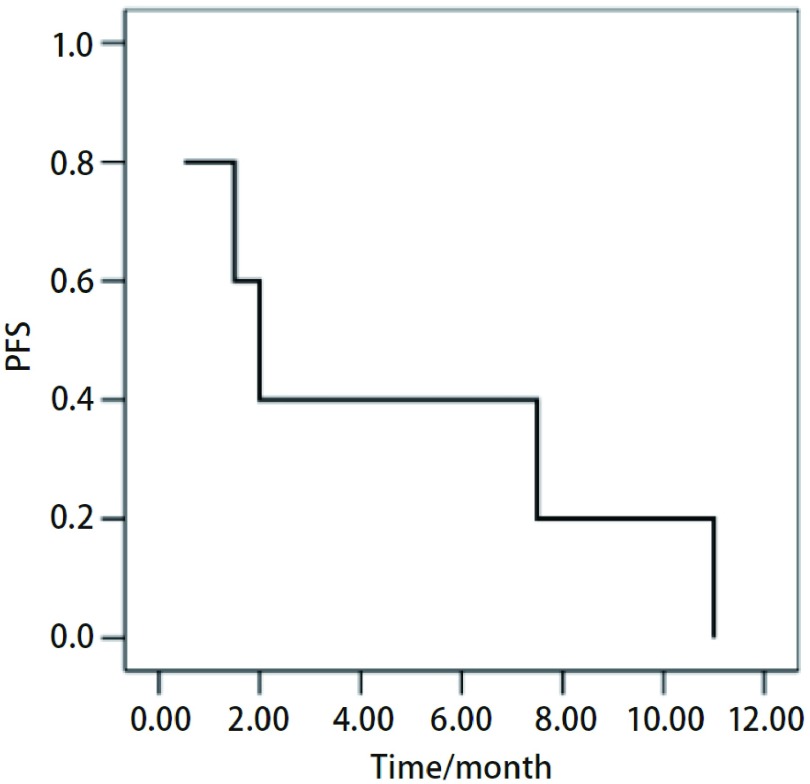
凡德他尼治疗肺腺癌患者的无进展生存曲线 *Kaplan*-*Meier* curve for progression free survival of lung adenocarcinoma patients treated by vandetanib

### 2.3 副反应

皮疹2例（40%），CTC分级均为Ⅰ级；皮肤改变（皮肤干燥、皲裂）2例（40%）：1例为Ⅰ级，另1例为Ⅲ级；甲沟炎2例（40%）：1例为Ⅰ级，另1例为Ⅲ级；出现无症状的心电图QTc延长2例（40%）：分级均为Ⅰ级；ST-T改变（ST波压低≤0.05 mV，T波低平改变）1例（20%），分级为Ⅰ级；腹泻1例（20%），分级为Ⅰ级；转氨酶增高1例（20%），分级为Ⅰ级（图 3）。

**3:**
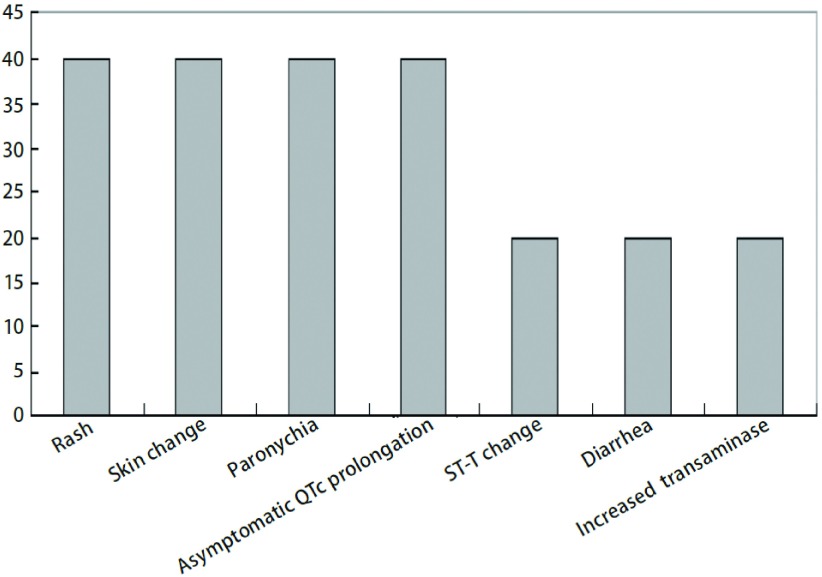
凡德他尼副反应的发生率（%） Rate of adverse events of vandetanib (%)

## 3 讨论

肺癌的治疗已从化疗时代转向个体化治疗，分子靶向治疗成为治疗晚期NSCLC的主要可供选择的方法，攻击肺癌细胞的靶点涉及到多个方面。EGFR和VEGF是目前两个研究较多的信号通路，也是多种恶性肿瘤生长和转移通路上的一个重要的步骤^[7, 8]^。

目前，单靶点靶向药物在临床上已经取得了一定的疗效，但肺癌是一个高度异质的疾病，信号传导是复杂的蛋白网络系统，抑制单一信号的传导不足以全部阻止肿瘤的发生发展，所以针对多靶点药物：舒尼替尼（sunitinib）、索拉非尼（sorafenib）、凡德他尼、阿法替尼（BIBW2992）、西地尼布（cediranib）等的研究越来越多；通过对多个肿瘤信号传导途径的抑制（这些途径可以作为救助或逃避的机制）导致抗肿瘤的协同作用^[6, 9]^，进一步提高靶向药物的治疗疗效。

凡德他尼（ZD6474, vandetanib, Zactima^TM^）是一种合成的苯胺喹唑啉化合物，口服的小分子多个细胞内受体激酶抑制剂^[7]^，能够抑制肿瘤细胞的生长、发展和血管生成（VEGFR-2，EGFR，酪氨酸激酶活性转染中的RET重排）过程中的不同的细胞内信号传导路径^[5]^。前期动物实验报导^[10]^，凡德他尼既可以抑制血管生成，还可抑制EGFR的传导。Ⅰ期临床研究^[11, 12]^显示凡德他尼≤300 mg的剂量能很好的耐受。

一项Ⅱ期随机研究^[13]^表明凡德他尼+PC化疗组较单一PC组治疗一线NSCLC PFS和OS均无明显改善。也有一些Ⅱ期研究评估显示凡德他尼在晚期肺癌治疗中PFS受益^[14]^。一项二线治疗晚期NSCLC的*meta*分析结果^[15]^提示，凡德他尼在晚期NSCLC的治疗中PFS和ORR获益，但OS无受益，毒性和标准二线化疗相似。ZODIAC^[16]^研究报道凡德他尼联合多西紫杉醇治疗较安慰剂联合多西紫杉醇治疗轻度提高PFS。与吉非替尼的对照研究^[17]^提示凡德他尼（11周）同吉非替尼（8.1周）相比，明显延长了PFS，两组之间OS无明显区别。

一项Ⅲ期试验^[18]^提示，凡德他尼联合培美曲塞化疗，对于培美曲塞单药二线治疗进展期NSCLC延长PFS无明显受益。但凡德他尼联合培美曲塞组客观缓解率（*P*＜0.001）和无症状恶化生存期（*P*=0.005, 2）具有明显优势。ZEST试验^[19]^显示凡德他尼和厄洛替尼相比，PFS（*P*=0.721）和OS（*P*=0.830）未体现出优越性，同时存在较多毒性反应。

皮疹、腹泻和高血压是凡德他尼的主要副反应^[13]^，具有良好的耐受性^[20]^。Natale的研究^[17]^发现，凡德他尼组3例出现3级高血压，未见严重出血或血栓，无症状的QTc间期延长发生率21%，而吉非替尼组为5%。ZODIAC研究^[16]^显示在3级或3级以上的毒副反应中，皮疹、嗜中性粒细胞减少、白细胞减少和发热性中性粒细胞减少症在凡德他尼组中更为常见，最常见的严重不良反应是发热性中性粒细胞减少，凡德他尼组占总不良反应发生率的7%，安慰剂组占6%。这可能同泰索帝的副反应相关。

本研究共5例患者，均已经过一线或二线的铂二联化疗，其中2例女性患者厄洛替尼靶向治疗时PFS分别为22个月和14个月，凡德他尼治疗时PFS分别为7.5个月和11个月，提示对于应用厄洛替尼治疗PFS长的患者应用凡德他尼PFS也长。第4、5例患者应用厄洛替尼治疗PFS短同时应用凡德他尼PFS也短，提示凡德他尼对于EGFR靶点的控制类似厄洛替尼，进而考虑EGFR-TKIs进展后选用其它药物或延迟一段时间再次使用EGFR-TKIs可能对于患者也具有一定的治疗意义。同时第4例患者为男性，第5例患者为女性，提示靶向治疗的敏感性并不能仅考虑性别因素，结合目前的研究结果，考虑EGFR-TKIs对于*EGFR*基因突变的患者更加敏感。第2例患者凡德他尼后PSF长于第1例患者，相反，厄洛替尼后PFS却短于第1例患者，说明在对*EGFR*基因作用的基础上，对于VEGF等其他靶点的控制也起到一定作用。第3例患者中的厄洛替尼后PFS为10个月，而凡德他尼后仅为1.5个月，说明凡德他尼对于本例患者的EGFR靶点的控制强于对于VEGF靶点的控制。

目前针对存在*EGFR*基因突变的肺腺癌患者进行TKIs的治疗，有效率较高，也获得了较长的PFS，但应用一段时间后，还是会出现肿瘤的复发和转移，具体的耐药机制还不明确。早期有研究^[21, 22]^证实EGFR T790M突变导致吉非替尼的耐药。T790M的点突变使EGFR能逃避吉非替尼的攻击^[23]^，所以应用多靶点药物进行抗肿瘤治疗覆盖面就会增大。对于体内存在T790M突变的，凡德他尼仍保持着明显的有效性^[24]^。

本研究的主要副反应以针对EGFR的皮肤粘膜损伤改变以及针对VEGF的血管生成方面的损伤为主。包括皮疹、皮肤反应、甲沟炎、腹泻、转氨酶增高，和无症状的心电图QTc延长、心电图ST-T改变，同当前临床研究的副反应相似^[14, 18]^。说明凡德他尼安全耐受性较好，未出现因毒副反应而终止治疗的患者，同IIa期剂量探索研究结果^[25]^。

综上所述，本研究中凡德他尼是针对5例三线或四线治疗的NSCLC患者，例数较少，在PFS上获益不大；分析原因一是与患者接收多程治疗，机体状况不佳有关；其次，患者已经出现某些基因或蛋白表达的改变、耐药而导致疗效不佳。遗憾的是本研究中的患者无条件进行基因突变的检测，故不能解释其疗效是否同基因突变存在相关性。还需要进一步的研究来评价凡德他尼在NSCLC治疗中的作用，确定合适的方案、选择合适的患者进行进一步的临床研究。
